# Supplementary material for: A multitask model for realtime fish detection and segmentation based on YOLOv5
Source: PeerJ Comput Sci. 2023 Mar 10;9:e1262. doi: 10.7717/peerj-cs.1262 (PMC10280594; doi:10.7717/peerj-cs.1262)
Supplement: Supplemental Information 3 [file peerj-cs-09-1262-s003.docx]

**Supplemental Table：**

**[The original source from where we obtained the third-party code](https://peerj.com/manuscripts/78416/declarations/#question_90) we used in our analysis.**

| **[third-party code](https://peerj.com/manuscripts/78416/declarations/#question_90)** | **the original source** |
| --- | --- |
| mmsegmentation | @misc{mmseg2020, title={{MMSegmentation}: OpenMMLab Semantic Segmentation Toolbox and Benchmark}, author={MMSegmentation Contributors}, howpublished = {\url{https://github.com/open-mmlab/mmsegmentation}}, year={2020} |
| mmdetection | @article{mmdetection, title = {{MMDetection}: Open MMLab Detection Toolbox and Benchmark}, author = {Chen, Kai and Wang, Jiaqi and Pang, Jiangmiao and Cao, Yuhang and Xiong, Yu and Li, Xiaoxiao and Sun, Shuyang and Feng, Wansen and Liu, Ziwei and Xu, Jiarui and Zhang, Zheng and Cheng, Dazhi and Zhu, Chenchen and Cheng, Tianheng and Zhao, Qijie and Li, Buyu and Lu, Xin and Zhu, Rui and Wu, Yue and Dai, Jifeng and Wang, Jingdong and Shi, Jianping and Ouyang, Wanli and Loy, Chen Change and Lin, Dahua}, journal= {arXiv preprint arXiv:1906.07155}, year={2019} } |
| YOLOv5 | URL={https://github.com/ultralytics/yolov5} |
